# Supplementary material for: Limited Supply of Protein and Lysine Is Prevalent among the Poorest Households in Malawi and Exacerbated by Low Protein Quality
Source: Nutrients. 2022 Jun 11;14(12):2430. doi: 10.3390/nu14122430 (PMC9230964; doi:10.3390/nu14122430)
Supplement: Supplementary file 1 [file nutrients-14-02430-s001.zip › Supplementary Data S2.pdf]

Limited supply of protein and lysine is prevalent among the poorest households in Malawi and exacerbated by low protein quality

Molly Muleya[1], Kevin Tang[2], Martin R. Broadley[1,3], Andrew Salter[1], Edward Joy[2,3]

[1]School of Biosciences, Future Food Beacon, University of Nottingham, Sutton Bonington Campus, Loughborough, Leicestershire LE12 5RD, UK

[2]Faculty of Epidemiology and Population Health, London School of Hygiene & Tropical Medicine, Keppel Street, London WC1E 7HT, UK

[3]Rothamsted Research, West Common, Harpenden, Hertfordshire, AL5 2JQ, UK

*Email: Molly.Muleya@nottingham.ac.uk

Tel: +44 (0) 115 95 16262

Supplementary data

Table S1: Estimated Average Requirements (EAR) of protein and indispensable amino acids for a 65 kg adult male

IAA	EAR (g kg^{-1} day^{-1})	EAR (g day^{-1})
Protein	0.660	42.9
AAA	0.025	1.625
His	0.010	0.650
Ile	0.020	1.300
Leu	0.039	2.535
Lys	0.030	1.950
SAA	0.015	0.975
Thr	0.015	0.975
Try	0.004	0.260
Val	0.026	1.690

AAA: Aromatic amino acids (phenylalanine + tyrosine), His – Histidine, Ile – isoleucine, Leu – Leucine, Lys – Lysine, SAA: Sulphur amino acids (methionine + cysteine), Thr – Threonine, Trp – Tryptophan. Estimates were derived from FAO (2011).

Table S2: Proportion of total protein and indispensable amino acid dietary supplies by food group

Food group	Protein	AAA	His	Ile	Leu	Lys	SAA	Thr	Trp	Val
Cereals	65.5%	67.9%	68.8%	61.8%	75.9%	44.3%	71.0%	63.8%	54.6%	66.9%
Legumes & nuts	14.9%	16.3%	15.4%	17.1%	11.3%	23.3%	11.2%	16.0%	20.5%	14.9%
Animal products	11.2%	9.8%	10.5%	14.2%	8.7%	23.2%	12.8%	13.5%	14.3%	11.6%
Vegetables	3.6%	2.8%	2.1%	3.0%	1.7%	4.0%	1.8%	2.9%	4.6%	2.7%
Roots & Tubers	1.9%	1.2%	1.2%	1.4%	0.8%	1.8%	1.5%	1.7%	3.1%	1.5%
Dairy	0.7%	0.8%	0.7%	1.0%	0.6%	1.4%	0.6%	0.8%	1.1%	0.9%
Fruits	0.4%	0.2%	0.4%	0.3%	0.2%	0.5%	0.2%	0.3%	0.5%	0.3%
Others	1.6%	0.9%	0.9%	1.2%	0.8%	1.4%	1.0%	1.1%	1.4%	1.1%

AAA: Aromatic amino acids (phenylalanine + tyrosine), His – Histidine, Ile – isoleucine, Leu – Leucine, Lys – Lysine, SAA: Sulphur amino acids (methionine + cysteine), Thr – Threonine, Trp – Tryptophan. 'Others' include foods from other food groups with minor contributions (less than 1%) such as beverages, fats & oils, spices & condiments and foods purchased from vendors.

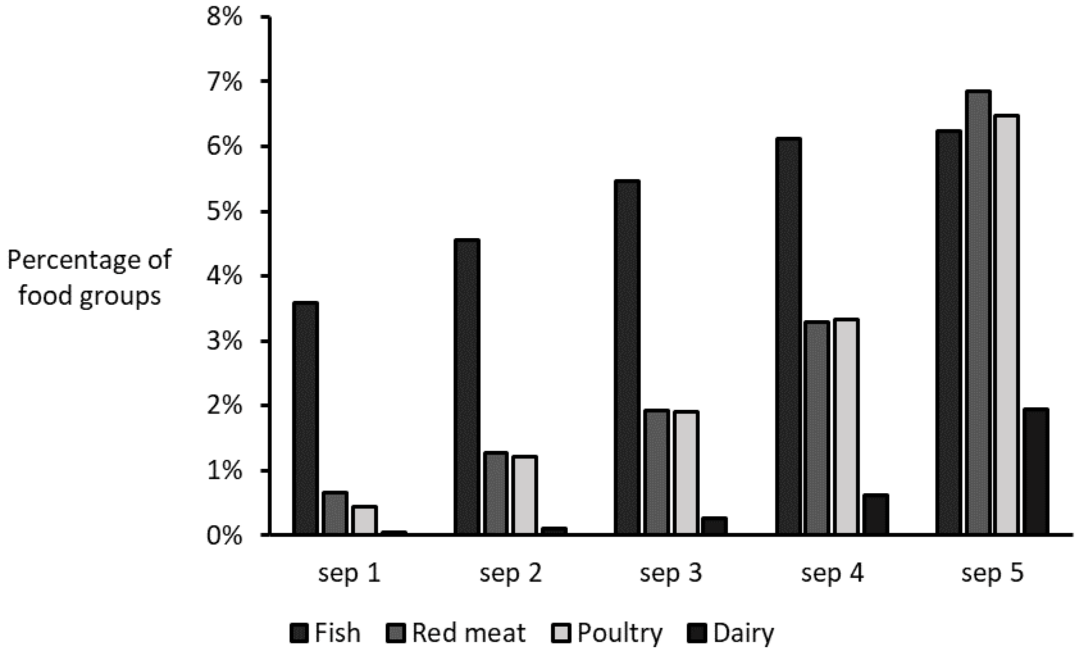

Figure S1: Proportion of households reporting consumption of animal products in the past 7-days, by household socioeconomic position

SEP: socioeconomic position (1 = poorest quintile, 5 = wealthiest quintile)

Table S3: Summary statistics of total and available indispensable amino acid dietary supplies

	Total supply (g AME^{-1} day^{-1})				Available supply (g AME^{-1} day^{-1})			
**Amino acid**	**Q1**	**Median**	**Q3**	**Mean**	**Q1**	**Median**	**Q3**	**Mean**
AAA	4.9	6.9	9.5	7.6	4.2	5.9	8.1	6.5
His	1.6	2.3	3.2	2.5	1.3	1.9	2.6	2.1
Ile	2.1	2.9	4.2	3.4	1.8	2.5	3.6	2.9
Leu	5.9	8.4	11.5	9.2	5.4	7.5	10.3	8.2
Lys	2.1	3.1	4.6	3.7	1.7	2.6	3.8	3.1
SAA	2.1	2.9	3.9	3.2	1.8	2.5	3.4	2.7
Thr	2.1	3.0	4.2	3.2	1.7	2.4	3.4	2.9
Try	0.5	0.7	0.9	0.8	0.4	0.6	0.8	0.6
Val	2.8	3.9	5.5	4.4	2.3	3.3	4.6	3.7

AAA: Aromatic amino acids (phenylalanine + tyrosine), His – Histidine, Ile – isoleucine, Leu – Leucine, Lys – Lysine, SAA: Sulphur amino acids (methionine + cysteine), Thr – Threonine, Trp – Tryptophan.

Table S4: Percentage of households at risk of indispensable amino acids deficiency due to inadequate dietary supply

IAA	Total supply	Available supply
Protein	12	21
AAA	2	3
His	3	4
Ile	7	11
Leu	3	4
Lys	21	33
SAA	4	5
Thr	3	6
Try	4	9
Val	7	11

AAA: Aromatic amino acids (phenylalanine + tyrosine), His – Histidine, Ile – isoleucine, Leu – Leucine, Lys – Lysine, SAA: Sulphur amino acids (methionine + cysteine), Thr – Threonine, Trp – Tryptophan.
